# Real-World Safety of Intravitreal Bevacizumab and Ranibizumab Treatments for Retinal Diseases in Thailand: A Prospective Observational Study

**DOI:** 10.1007/s40261-018-0678-5

**Published:** 2018-08-01

**Authors:** Sermsiri Sangroongruangsri, Usa Chaikledkaew, Suthasinee Kumluang, Olivia Wu, Claudia Geue, Tanapat Ratanapakorn, Pattara Leelahavarong, Lily Ingsrisawang, Paisan Ruamviboonsuk, Wongsiri Taweebanjongsin, Janejit Choovuthayakorn, Apichart Singalavanija, Prut Hanutsaha, Kittisak Kulvichit, Thitiporn Ratanapojnard, Warapat Wongsawad, Yot Teerawattananon

**Affiliations:** 1 Social and Administrative Pharmacy Excellence Research (SAPER) Unit, Department of Pharmacy, Faculty of Pharmacy, Mahidol University, 447 Sri-Ayuthaya Road, Rajathevi, Bangkok, 10400 Thailand; 2 Health Intervention and Technology Assessment Program, Ministry of Public Health, Nonthaburi, Thailand; 3 Institute of Health and Wellbeing, University of Glasgow, Glasgow, UK; 4 Department of Ophthalmology, Khon Kaen University, Khon Kaen, Thailand; 5 Department of Statistics, Faculty of Science, Kasetsart University, Bangkok, Thailand; 6 Department of Ophthalmology, Faculty of Medicine, Rajavithi Hospital, Rangsit University, Bangkok, Thailand; 7 Mettapracharak Eye Institute, Mettapracharak (Wat Rai Khing) Hospital, Nakhon Pathom, Thailand; 8 Department of Ophthalmology, Faculty of Medicine, Chiang Mai University, Chiang Mai, Thailand; 9 Department of Ophthalmology, Faculty of Medicine Siriraj Hospital, Mahidol University, Bangkok, Thailand; 10 Department of Ophthalmology, Ramathibodi Hospital, Mahidol University, Bangkok, Thailand; 11 Department of Ophthalmology, Faculty of Medicine, Chulalongkorn University, Bangkok, Thailand; 12 Department of Ophthalmology, Phramongkutklao Hospital, Phramongkutklao College of Medicine, Bangkok, Thailand

## Abstract

**Background:**

There is very limited evidence examining serious systemic adverse events (SSAEs) and post-injection endophthalmitis of intravitreal bevacizumab (IVB) and intravitreal ranibizumab (IVR) treatments in Thailand and low- and middle-income countries. Moreover, findings from the existing trials might have limited generalizability to certain populations and rare SSAEs.

**Objectives:**

This prospective observational study aimed to assess and compare the safety profiles of IVB and IVR in patients with retinal diseases in Thailand.

**Methods:**

Between 2013 and 2015, 6354 patients eligible for IVB or IVR were recruited from eight hospitals. Main outcomes measures were prevalence and risk of SSAEs, mortality, and endophthalmitis during the 6-month follow-up period.

**Results:**

In the IVB and IVR groups, 94 and 6% of patients participated, respectively. The rates of outcomes in the IVB group were slightly greater than in the IVR group. All-cause mortality rates in the IVB and IVR groups were 1.10 and 0.53%, respectively. Prevalence rates of endophthalmitis and non-fatal strokes in the IVB group were 0.04% of 16,421 injections and 0.27% of 5975 patients, respectively, whereas none of these events were identified in the IVR group. There were no differences between the two groups in the risks of mortality, arteriothrombotic events (ATE), and non-fatal heart failure (HF). Adjustment for potential confounding factors and selection bias using multivariable models for time-to-event outcomes and propensity scores did not alter the results.

**Conclusions:**

The rates of SAEs in both groups were low. The IVB and IVR treatments were not associated with significant risks of mortality, ATE, and non-fatal HF.

**Trial Registration:**

Thai Clinical Trial Registry identifier TCTR20141002001.

**Electronic supplementary material:**

The online version of this article (10.1007/s40261-018-0678-5) contains supplementary material, which is available to authorized users.

## Key Points

**Table:** 

Safety evidence of IVB and IVR derived from randomized controlled trials and studies in other settings might not be generalizable to the Thai population in routine clinical practice.
This is the first large prospective observational study examining the safety of IVB compared with IVR in Thailand and low- and middle-income countries (LMICs) where there is a lack of appropriate infrastructure for repackaging the pre-filled bevacizumab syringes compared to high-income countries.
This study found low rates of pre-specified serious systemic adverse events and endophthalmitis, which are consistent with the studies conducted in developed countries in terms of the short-term safety profiles of IVB and IVR

## Introduction

Many retinal diseases are leading causes of visual disability worldwide [1, 2]. The global pooled prevalence estimates derived from population-based studies for age-related macular degeneration (AMD), diabetic macular edema (DME), and retinal vein occlusion (RVO) were 8.69% of people aged 30–97 years [3], 7.48% of diabetic patients aged 20–79 years [4], and 0.52% of people worldwide [5], respectively. The rapid growth of an aging population and the rising impact of non-communicable diseases have led to a substantial increase in the number of patients suffering from these retinal disorders. Asia is the most populous continent in the world, and it was estimated that nearly one-third of patients affected by AMD will reside in this region by 2040 [3]. Poor vision from central vision loss leads to devastating consequences for the affected patients, their families, and society in terms of economic burden, quality of life, carrying out daily activities, including work, and psychosocial impacts [6–8]. Access to effective treatments in a timely manner is crucial to counteract the burden of visual loss due to such diseases.

During the past decade, vascular endothelial growth factor (VEGF) inhibitors have played an essential role in preserving and restoring visual impairment from a variety of retinal conditions. The off-label use of intravitreal bevacizumab (IVB) was prevalent before ranibizumab was licensed for nAMD in 2006 [9]. Moreover, limited access to intravitreal ranibizumab (IVR) therapy due to its high cost has driven the off-label use of IVB in many countries [10–12]. In Thai public hospitals, the cost of a single dose of ranibizumab was US$1371 (the reference price in 2015 from the Drug and Medical Supply Information Center, Thailand) while the minimum cost of pre-filled bevacizumab syringes for intravitreal injections (IVT) was approximately US$23.

Improper compounding procedures in repackaged bevacizumab for IVT have been associated with microbial contamination causing post-injection endophthalmitis outbreaks [13–15]. This issue is problematic, particularly among low- and middle-income countries (LMICs) where there is a lack of appropriate infrastructure for repackaging the pre-filled syringes compared to high-income countries (HICs). Another concern [which has been reported from the use of intravenous (IV) bevacizumab for cancer treatments] is the occurrence of rare serious systemic adverse events (SSAEs) in relation to its ability to inhibit the VEGF pathway, notably thromboembolic events [16–21]. A possible association between anti-VEGF therapy and SSAEs, particularly the risks of arterial thromboembolic events (ATEs), systemic hemorrhage, heart failure (HF), venous thromboembolism (VTE), hypertension (HT), and vascular death, has been raised [22]. However, previous studies [16, 17, 23, 24] found no statistically significant differences between the IVB and IVR therapies in terms of the occurrence of these SSAEs, including ATEs and death. Even though IVB is considered to be comparable to IVR in terms of effectiveness and safety for ocular treatment [10, 12, 19, 22, 25–30] at a much lower price, difficulties have arisen from its off-label use from a legal aspect when the licensed drug is available [31, 32], and debates over the validity and methodological limitations of existing drug safety evidence [16, 17, 23, 33].

Most Thai people are eligible for necessary health services according to the three main national health insurance (NHI) schemes under the universal health coverage policy: the Civil Servant Medical Benefit Scheme (CSMBS), the Universal Coverage scheme (UC), and the Social Security Scheme (SSS) [34]. The CSMBS—which provides healthcare benefits to government employees and their dependents, and retirees, and comprises approximately 8% of the Thai population—has a policy which restricts reimbursement for off-label medicines. Therefore, ranibizumab, which was the only FDA-approved anti-VEGF drug for ophthalmic indications available and licensed in 2007, was included in the benefit package of the CSMBS. As a result, CSMBS beneficiaries had access to high-priced IVR free of charge. However, none of the anti-VEGF drugs were listed in the National List of Essential Medicines (NLEM) for patients with retinal diseases in the other NHI schemes (the UC and SSS) prior to 2012.

Comparative effectiveness research [19] showed that IVB and IVR had equal efficacy for treatments of neovascular age-related macular degeneration (nAMD) and DME, but safety was inconclusive due to insufficient evidence. Based on the findings of this study, the multiple-stakeholder panel suggested that ranibizumab was the preferable choice if the producer agreed to reduce the price; otherwise, the subcommittee of the NLEM should include bevacizumab instead of ranibizumab and develop a drug safety monitoring system instead. Subsequently, in 2012, IVB was included in the NLEM for treating nAMD and DME after negotiations to reduce the price of ranibizumab failed [35]. Since then, access to IVB therapy for nAMD and DME for Thai beneficiaries under the UC and SSS, which represents 92% of the Thai population, has been enhanced. Eight months after the policy action on the off-label use of IVB in Thailand, IVB was listed in a complementary list of the 18th WHO Model List of Essential Medicines (EML) for nAMD [36]. This decision was made taking into consideration the available evidence of benefit-risk profiles between IVB and IVR, and public health need for affordable nAMD treatment.

Evidence assessing the safety of pre-filled syringes of bevacizumab for macular disease treatment in LMICs and Thailand was scarce. Additionally, there was limited evidence comparing the risks of serious adverse events (SAEs) between IVB and IVR treatments to support policy decision making. A few studies [37–39] were conducted in the Thai setting to assess the safety of IVB and IVR. All were retrospective medical chart reviews with small sample sizes. Thus, this team opted to determine the safety of these drugs in real-world settings and to supplement findings from previous randomized controlled trials (RCTs), which had limited generalizability.

Therefore, the study aimed to determine the prevalence of SAEs of interest and to compare those risks between patients treated with IVB and IVR using real-life data from the Thai clinical setting.

## Methods

### Study Design

This study was designed as a prospective, multicenter, observational study conducted in patients with retinal vascular diseases who underwent either IVB (1.25 mg/0.05 mL) or IVR (0.5 mg/0.05 mL) treatments. Using convenience sampling [40], participants were followed up for 6 months after enrollment where each person underwent ocular examinations and treatments according to routine clinical practices. This study was registered with the Thai Clinical Trials Registry (TCTR20141002001).

### Study Setting and Participants

This study started a few months after the initiation of reimbursing IVB for patients with nAMD and DME under the NLEM policy. Patients with retinal diseases were prospectively recruited from the outpatient ophthalmology clinics of eight tertiary and teaching hospitals located in Central, Northern, and North-eastern Thailand between January 2013 and August 2014. These medical centers were chosen for their capacity to diagnose and treat retinal disorders with IVB and IVR; this included diagnostic equipment such as fundus fluorescein angiography (FFA) and optical coherence tomography (OCT), operating theatres, experienced retinal specialists, and other physicians who could deal with any complications arising from retinal diseases or anti-VEGF therapy. The pharmacy department in each hospital was responsible for the repackaging of IVB in bulk in laminar flow clean rooms using sterile techniques, as with drugs for intravenous injection or cytotoxic drugs. Containers were labelled with drug name, dates of preparation and expiration, preparation lot number, shelf-life, and storage condition. These prefilled syringes were refrigerated before administration and containers with ice or gel packs were used during the drug transfer. The repackaged bevacizumab must be used in 14 to 30 days, as indicated on the label.

#### Inclusion Criteria

Regardless of sex, patients aged ≥ 18 years who were either IVB- or IVR-naïve or had previously received IVB or IVR treatment for retinal pathologies, were eligible to participate. All participants were required to provide written informed consent at the time of enrollment.

#### Exclusion Criteria

Patients were excluded from the study if they had been treated with IVB within the 6 months prior to participation in the IVR-treated group at enrollment, and vice versa. This ensured that there was no interference effect from the other drug.

A safety analysis was performed only on datasets from participants with at least one post-baseline assessment.

### Data Collection

Patient interviews and medical record reviews were conducted at least once a month for a period of 6 months. Safety checks were performed via telephone calls when patients missed scheduled clinic visits for longer than a month to ask if they had experienced any SAEs since the previous visit. These patient data were documented in the designed paper case report forms (pCRFs). Afterwards, the pCRFs were reviewed and corrected to ensure accuracy and completeness before undergoing independent double data entry and data validation. Any discrepancies in the database, particularly key baseline characteristics and diagnosis of events of interest, were reconciled against the original pCRFs or medical records; a third party was used if necessary.

We also used data from the two national databases. The National Health Security Office (NHSO)’s in-patient database provides hospital admission data of beneficiaries under the three NHI schemes—which cover more than 90% of all admissions from public hospitals and contracted private hospitals in Thailand. The other source of data from the Bureau of Health Policy and Strategy (BPS), Ministry of Public Health was used to obtain the mortality data of Thai citizens from the Ministry of Interior’s civil registration database. The data from these two databases helped to identify hospitalization due to SAEs, or death occurring at hospitals outside of our study sites as well as death outside of hospitals during the follow-up period for all patients, including those who were lost to follow-up. The medical records of in-hospital deaths were independently reviewed by two physicians to verify causes of death; possible discrepancies were resolved by a third physician. When deaths could not be verified due to insufficient information or lack of access to medical records, the cause of death as indicated in the death certificate was applied.

### Variables, Interventions Exposures, and Outcomes

At the enrollment date, demographic and socioeconomic data, comorbidities, relevant medical history, smoking status, concomitant anticoagulant and antiplatelet therapies, history of IVB or IVR treatment, indication for IVB or IVR therapy, and ocular assessments were recorded as baseline characteristics. Since the selection of health interventions of each patient in Thai public hospitals relies heavily on the NHI scheme rather than other socioeconomic factors, we used the NHI scheme as a covariate representing the socioeconomic status (SES) of patients and categorized it into CSMBS and non-CSMBS (included the UC and the SSS subgroups).

Treatment information, such as the number of treated eyes, number of IVR and IVB injections, timing of each injection, use of topical antibiotics after IVT, room for IVT administration (i.e. operating room or other rooms), date and lot number of repackaged bevacizumab for ocular use, and IVT of other drugs, were recorded.

Primary outcomes of interest were the prevalence rates of non-fatal strokes and endophthalmitis in the group receiving IVB. Secondary outcomes were SSAEs proposed by previous studies, which included non-fatal ischemic heart disease (IHD) or myocardial infarction (MI), ATE, VTE, HF, transient ischemic attack (TIA), gastrointestinal (GI) hemorrhage or perforation, all-cause mortality, and death from vascular-related causes. ATE was defined as the occurrence of a non-fatal stroke, non-fatal MI or IHD. Vascular death included deaths after a stroke, MI/IHD, or cardiac arrest, and VTE comprised pulmonary embolism (PE) and deep vein thrombosis (DVT) [41].

### Statistical Analysis

Descriptive statistics was used to describe demographics, baseline characteristics, and safety outcomes; the mean and standard deviations (SD) were used for continuous variables. Frequencies and percentages were used for describing categorical variables. The unit of analysis for SSAEs was per person, while the rate of ocular SAEs was reported as per total patients, total treated-eyes, and total drug injections.

For SSAEs, incidence rates were calculated with event-free probabilities and were illustrated as Kaplan–Meier (K–M) curves (Online Resource 1). Data about individuals were censored at the time of loss to follow-up, consented withdrawal, drug switching from initial treatment to another, being event-free at the end of follow-up period, or death (in the case of non-fatal outcomes)—whichever came first. Univariable and multivariable time-to-event analyses were used to identify risk factors associated with systemic safety outcomes as well as to compare risk of SSAEs between the IVB and IVR groups. We assumed that patients went missing at random and treatment allocation between IVB and IVR is independent of the outcome of interest. Missing data were not imputed.

As the rates of specific SAEs were expected to be low, these SAEs were combined as composite outcomes. Only all-cause mortality, ATE, and non-fatal HF were assessed in time-to-event analyses. Moreover, some covariates were combined with the aim of achieving at least 5–10 events per variable (EPV) to minimize bias and variability of estimates and unreliable results [42, 43]. Covariates included were: age at enrollment, sex (female vs male), NHI (non-CSMBS vs CSMBS), comorbidity related to CV risk factors [number of the following comorbidities: diabetes (DM), HT, dyslipidemia (DLP), chronic kidney disease (CKD), IHD, and stroke], use of antiplatelet drugs (no vs yes), and groups of total number of intravitreal drug injections in any eyes before the end of the study (1–3 injections vs > 3 injections). Possible interactions between covariates were also considered in the model when the number of the events became sufficient.

The propensity score (PS) method was applied to minimize selection bias due to the imbalance of measured baseline covariates between the IVB and IVR groups. The PS is the probability of a participant being treated with a certain treatment given a set of covariates [44, 45]. The PS was calculated by age group (aged 18–51, 52–59, and 60–97 years), sex, DM, HT, IHD, stroke, and CKD. The PS was included as a continuous covariate in the multivariable time-to-event models.

Multiple models were constructed to determine the best fit for the data. These models included the multivariable Cox proportional hazards regression model, the Weibull proportional hazards model, Exponential model, and Gompertz model. Model adequacy for each model and the proportionality assumption for Cox proportional hazards regression model were also assessed. Ultimately, the final models were chosen based on the lowest Akaike Information Criterion (AIC) value as that would suggest a better fit for the data [46]. The results from the selected models are presented as unadjusted and adjusted hazard ratios (aHR) with a 95% confidence interval (95% CI).

All statistical analyses were performed with STATA version 14.2 (StataCorp, College Station, TX), using a two-sided statistical significance level of 0.05.

## Results

### Participants

**Fig. 1:**
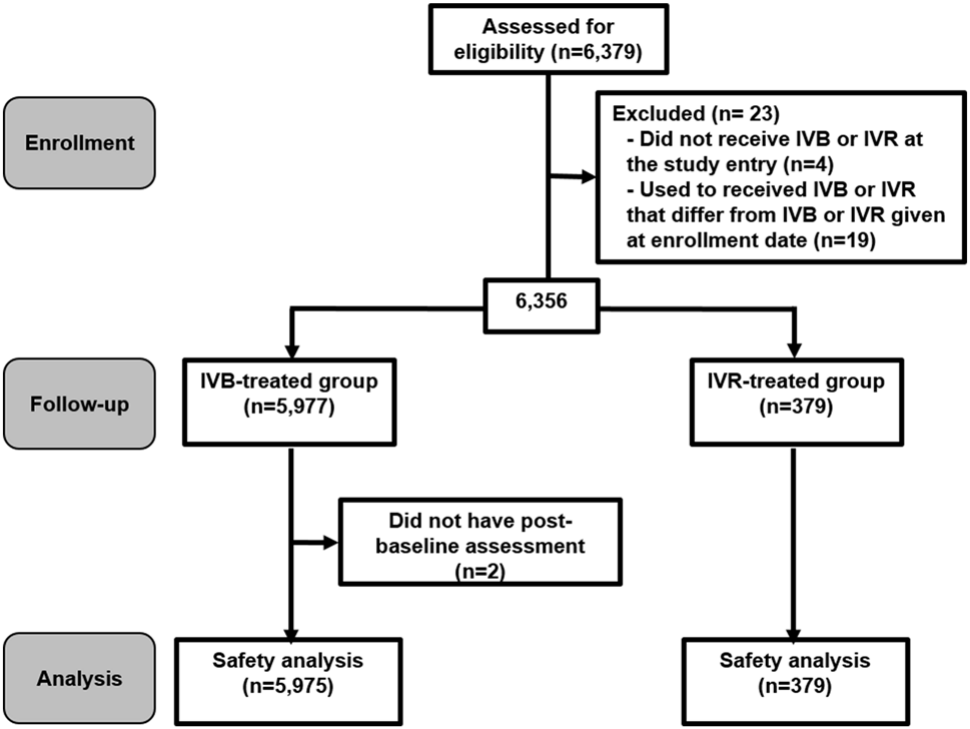
Flow diagram of patient participation

A total of 6379 patients were initially recruited (Fig. 1). However, four patients were excluded because they did not receive either IVB or IVR treatment on the enrollment date, but the reasons were not recorded. Nineteen patients with a history of drug switching within 6 months before the study enrollment were also excluded (14 and 5 patients had previously been treated with IVB and IVR before receiving IVR and IVB at enrollment, respectively). This reduced the total number to 6356 patients. Finally, two patients without post-baseline safety assessments were also excluded. As a result, the final analysis population consisted of 5975 patients in the IVB group and 379 patients in the IVR group.

### Patient Characteristics

Demographics and baseline characteristics of the study cohort based on the drug groups are summarized in Table 1. Patients aged ≥ 65 years accounted for 30.1% of the cohort. Overall, patients in the IVR group were older than those in the IVB group. In the IVR group, the proportion of patients under the CSMBS was larger than patients under the UC and SSS. Other significant differences can be seen in the proportion of patients with DM or CKD (Online Resource 1) and the use of antiplatelet drugs, which was greater in the IVB group than the IVR group. There was also a higher proportion of IVR-treated patients who were diagnosed with IHD or stroke than in the IVB-treated group.

**Table 1:** Demographics and baseline characteristics by treatment group

Demographic variables	Ranibizumab (*n* = 379)		Bevacizumab (*n* = 5975)
Age (years)			
Mean age ± SD	66 ± 13		58 ± 12
Aged < 65	159 (42.0%)		4238 (70.9%)
Aged ≥ 65 (elderly)	220 (58.0%)		1715 (28.7%)
Missing values	0		22 (0.4%)
Sex			
Female	172 (45.4%)		3182 (53.3%)
Male	207 (54.6%)		2793 (46.7%)
NHI			
Non-CSMBS	109 (28.8%)		4558 (76.3%)
CSMBS	178 (47.0%)		783 (13.1%)
Missing values	92 (24.3%)		634 (10.6%)
Smoking status			
Non-smokers	342 (90.2%)		5382 (90.1%)
Smokers	35 (9.2%)		580 (9.7%)
Missing values	2 (0.5%)		13 (0.2%)
Comorbidities			
No comorbid condition	79 (20.8%)		796 (13.3%)
Had at least one comorbidity	300 (79.2%)		5179 (86.7%)
Mean ± SD (conditions^a^)	2 ± 1		2 ± 1
Concomitant medicines			
Anticoagulants			
No	369 (97.4%)		5887 (98.5%)
Yes	7 (1.9%)		66 (1.1%)
Missing values	3 (0.8%)		22 (0.4%)
Antiplatelet drugs			
No	247 (65.2%)		3537 (59.2%)
Yes	129 (34.0%)		2413 (40.4%)
Missing values	3 (0.8%)		25 (0.4%)
History of IVR and IVB treatment			
IVB and IVR naïve	160 (42.2%)		3096 (51.8%)
IVB or IVR experienced	219 (57.8%)		2879 (48.2%)
Recent stroke^b^			
No	379 (100.00%)		5974 (99.98%)
Yes	0		1 (0.02%)
Recent endophthalmitis^b^			
No	379 (100.00%)		5973 (99.97%)
Yes	0		2 (0.03%)
Retinal diseases (treated-eye)^c^			
nAMD and CNV	136 (32.7%)		666 (8.8%)
PCV	103 (24.8%)		641 (8.4%)
DME	50 (12.0%)		2896 (38.1%)
RVO	66 (15.9%)		808 (10.6%)
PDR and related complications	37 (8.9%)		1891 (24.8%)
Others	24 (5.8%)		706 (9.3%)
Missing values	0 (0.0%)		3 (0.04%)

Data are presented as number (percentage) unless otherwise indicated

Age variable had a skewed distribution which median ages (25th–75th percentile) for IVR and IVB groups were 68 (20–97) and 58 (18–94) years respectively. Non-CSMBS group included patients under the Universal Health Coverage Scheme (UC) and Social Security Scheme (SSS)

*CNV* choroidal neovascularization, *CSMBS* Civil Servant Medical Benefit Scheme, *DME* diabetic macular edema, *IVB* intravitreal bevacizumab, *IVR* intravitreal ranibizumab, *nAMD* neovascular age-related macular degeneration, *NHI* National Health Insurance scheme, *PCV* polypoidal choroidal vasculopathy, *PDR* proliferative diabetic retinopathy, *RVO* retinal vein occlusion

^a^Number of the following comorbid conditions: diabetes, hypertension, dyslipidemia, chronic kidney disease, ischemic heart disease, and stroke

^b^Recent stroke or endophthalmitis were defined as a hospitalization due to stroke or endophthalmitis within 6 months prior to enrollment date

^c^Total 8027 treated-eyes (IVR = 416 versus IVB = 7611)

### Treatment Pattern

Most patients received unilateral treatment during the 6-month follow-up period (90.2 and 72.6% in the IVR and IVB groups, respectively), and about half of patients had previously received IVR or IVB treatment. The mean follow-up times and standard deviation (SD) from the enrollment date until the end of the follow-up period, or death or drug switching of patients enrolled in the IVR and IVB groups were 161 ± 42 and 172 ± 29 days, respectively. The proportion of patients who switched drugs from IVR to IVB (73 of 379) was much higher than patients who switched from IVB to IVR (192 of 5975) during the entire follow-up. The rates of loss to follow-up were similar across both groups: 565 of 5975 (9.5%) in the IVB group and 38 of 379 (10.0%) in the IVR group.

The mean ± SD of total drug injections during the follow-up period of the IVR and IVB groups were 2.6 ± 1.6 and 2.7 ± 1.9 injections, respectively, and the total number of injections in the IVR and IVB groups were 974 and 16,421 injections, respectively. The number of topical antibiotics used after intravitreal injection was 932 (95.7% of total IVR injections) and 15,770 (96.0% of total IVB injections). The IVTs were performed at operating rooms for 10% of total injections (IVR = 73 vs IVB = 1671 injections) and non-operating rooms including outpatient clinics for 90% of total injections (IVR = 893 vs IVB = 14,698 injections).

### Ocular Event: Endophthalmitis

There were six treated eyes (0.08%) from six patients in the IVB group who contracted endophthalmitis, while there were no events observed in the IVR group. This serious ocular event developed within 1 month of intravitreal injection of the affected eyes in 4 out of the 6 patients (mean ± SD 4.25 ± 0.96 days, or approximately 3–5 days). The fifth patient was diagnosed with postoperative endophthalmitis from cataract surgery, which occurred 73 days after IVB injection. The last case was admitted due to occurrence of endophthalmitis 49 days after the IVB injection. In three of the six patients, visual acuity was 20/40, 20/100, and 6/12 prior to developing endophthalmitis. However, even though they received endophthalmitis therapy, visual acuity for these three eventually declined to no light perception (NLP).

Results from the gram-stain and microbiological culture of vitreous fluid were available for only three patients who developed endophthalmitis. Gram-stain revealed gram-positive cocci for these three cases. The vitreous cultures identified coagulase-negative Staphylococci for the two cases while the result was not reported for one case.

### SSAEs

Non-fatal strokes occurred in only 16 of the 5975 patients (0.27%) in the IVB group. The overall rate of mortality from any cause was 1.07%, which comprised 2 of the 379 IVR-treated patients (0.53%) and 66 of the 5975 IVB-treated patients (1.10%). The rates of individual SAEs of interest during the 6-month follow-up period ranged from 0 to 1.21%; rates between the drug groups were similar (Table 2). Among SAEs of interest, there were only two patients in the IVR group who experienced acute subendocardial myocardial infarction and congestive heart failure (CHF), respectively. Non-fatal MI or IHD occurred in 20 patients (0.33%) in the IVB group. The proportions of patients who experienced vascular death, cardiovascular events (ATE or HF), VTE, and one or more non-fatal SSAEs in the IVB group were higher than the rates of the IVR group.

**Table 2:** Serious adverse events within 6 months of enrollment

Safety events	Ranibizumab (*n* = 379)		Bevacizumab (*n* = 5975)	
	Number of events	Affected patients	Number of events	Affected patients
Serious systemic events				
All-cause mortality	2	2 (0.53%)	66	66 (1.10%)
Arterial thrombotic events^a^	1	1 (0.26%)	57	46 (0.77%)
Non-fatal MI/IHD	1	1 (0.26%)	27	20 (0.33%)
Non-fatal stroke	0	0 (0.00%)	17	16 (0.27%)
Death from vascular causes^a^	0	0 (0.00%)	13	13 (0.22%)
Arterial thrombotic events or heart failure^a^	2	2 (0.53%)	146	111 (1.86%)
Heart failure	1	1 (0.26%)	89	72 (1.21%)
Venous thrombotic events^a^	0	0 (0.00%)	1	1 (0.02%)
Pulmonary embolism	0	0 (0.00%)	1	1 (0.02%)
Hospital admission for GI hemorrhage /perforation	0	0 (0.00%)	6	6 (0.10%)
Transient ischemic attack	0	0 (0.00%)	2	2 (0.03%)
≥ 1 non-fatal serious systemic events^a^	–	2 (0.53%)	–	109 (1.82%)
Serious ocular event: endophthalmitis				
Per persons	0	0 (0.00%)	6	6 (0.10%)
Per treated-eyes^b^	0	0 (0.00%)	6	6 (0.08%)
Per injection^c^	0	0 (0.00%)	6	6 (0.04%)

Data were presented as number (percentage). Events in the same SAE category of each affected patient were counted only once. Vascular death comprises deaths due to stroke, IHD, and MI. Arterial thrombotic event defined as patients who experienced non-fatal MI/IHD, non-fatal stroke, or vascular death

*GI* gastrointestinal, *IHD* ischemic heart disease, *MI* myocardial infarction

^a^Composite outcomes

^b^Treated-eyes of IVR and IVB were 416 and 7611, respectively

^c^Total number of IVT during 6 months and have no drug switching was 17,395 (IVR 974 and IVB 16,421 injections)

There was no significant increase in rates of all-cause mortality, ATE, and non-fatal HF when comparing the risk profiles between patients treated with IVB and IVR. The unadjusted HR (95% CI) for risks of all-cause mortality, ATE, and non-fatal HF in the treatment groups were 1.96 (95% CI 0.48–8.01), 2.70 (95% CI 0.37–19.60), and 4.23 (95% CI 0.59–30.42), respectively.

The final multivariable models of the outcomes considered only the main effects to avoid the risk of overfitting when including interactions as well as variables with very low counts. After adjustment, the aHRs were not statistically significantly different between the IVB and IVR groups (Table 3). The aHR values of the treatment group for all-cause mortality, ATE, and non-fatal HF were 1.8 (95% CI 0.2–13.2), 1.7 (95% CI 0.2–12.5), and 1.4 (95% CI 0.2–9.9), respectively. Patients with a higher number of comorbid conditions experienced increased risks of all-cause mortality (aHR 1.6; 95% CI 1.2–2.0), ATE (aHR 2.1; 95% CI 1.6–2.8), and non-fatal HF (aHR 2.2; 95% CI 1.7–2.8). The risk of ATE in male patients was 2.5 times higher (aHR 2.5; 95% CI 1.3–4.7) than for female patients. In the case of non-fatal HF, CSMBS beneficiaries had lower risk than patients from other NHI schemes (aHR 0.2; 95% CI 0.1–0.8). Patients who received a higher number of injections (> 3 injections) had statistically significantly lower risks of all-cause mortality (aHR 0.08; 95% CI 0.02–0.34) and ATE (aHR 0.25; 95% CI 0.09–0.69) than those treated with ≤ 3 injections.

**Table 3:** Hazard ratios of patients who received IVB versus IVR treatment after adjusting for covariates and propensity scores

Covariate^a^	All-cause mortality^b^				ATE^c^				Non-fatal HF^c^			
	Coef.	SE	aHR	95% CI	Coef.	SE	aHR	95% CI	Coef.	SE	aHR	95% CI
Treatment												
IVR vs IVB	0.58	1.02	1.78	0.24–13.18	0.51	1.03	1.66	0.22–12.47	0.30	1.01	1.35	0.19–9.88
Age (years)^e^	0.02	0.02	1.02	0.98–1.05	− 0.02	0.02	0.98	0.95–1.02	− 0.02	0.01	0.98	0.96–1.01
Sex												
Female vs male	0.48	0.26	1.62	0.97–2.69	0.90	0.33	2.46	1.29–4.67	− 0.22	0.25	0.80	0.49–1.30
NHI												
Others vs CSMBS	− 0.22	0.41	0.80	0.36–1.80	− 1.07	0.61	0.34	0.10–1.14	− 1.63	0.72	0.20	0.05–0.81
Total drug injections												
1–3 vs > 3 injections	− 2.48	0.72	0.08	0.02–0.34	− 1.40	0.52	0.25	0.09–0.69	− 0.47	0.30	0.62	0.35–1.12
Comorbidities^d,e^ (conditions)	0.44	0.13	1.55	1.19–2.01	0.75	0.15	2.12	1.59–2.84	0.77	0.13	2.17	1.69–2.78
Use antiplatelet drugs												
No vs yes	0.03	0.27	1.03	0.61–1.75	0.60	0.35	1.82	0.91–3.64	0.48	0.27	1.61	0.95–2.73
Propensity score^e^	19.27	7.17			− 7.23	3.81			5.61	5.73		
Constant	− 30.63	7.47			− 7.01	4.38			− 19.36	6.08		

*aHR* adjusted hazard ratio, *ATE* arterial thrombotic event, *CI* confidence interval, *Coef.* standardized coefficient, *CSMBS* Civil Servant Medical Benefit Scheme, *HF* heart failure, *IVB* intravitreal bevacizumab, *IVR* intravitreal ranibizumab, *NHI* National health insurance scheme, *SE* standard error

^a^Covariate reference groups: IVR, female, other health insurance scheme (UC and SSS), 1–3 injections, and not use antiplatelet drugs

^b^Exponential model

^c^Weibull model

^d^Number of the following comorbid conditions: diabetes, hypertension, dyslipidemia, chronic kidney disease, ischemic heart disease, and stroke

^e^Continuous variable

## Discussion

### Principal Findings

While off-label IVB may be a preferable alternative option to IVR in Thailand and LMICs, there were very few studies which have attempted to evaluate the safety of IVB treatment in these countries. Additionally, there was an insufficient number of large databases able to generate evidence through routine practice. To the best of our knowledge, this is the first large prospective observational study examining the safety of IVB compared with IVR in these settings. We primarily aimed to generate a safety profile of off-label IVB as part of a policy to establish a safety monitoring system after the inclusion of this medication into the NLEM. The results of this study supported the short-term safety of the off-label use of IVB in Thailand. Overall, the prevalence rates of systemic events and endophthalmitis were infrequent (ranging from 0 to 1.21%). Cardiovascular events accounted for the largest proportion among non-fatal SSAEs of interest in both drug groups (HF: IVB 1.21% vs IVR 0.26%, and IHD/MI: IVB 0.33% vs IVR 0.26%). We found that more than 80% of these cases had several comorbid conditions including IHD, and this confounder was adjusted in the multivariable models. Although the rate of the ocular SAE was lower than more severe SSAEs, low rates of SAEs could be seen in the entire cohort and were similar in both drug groups. We found that the IVB treatment did not significantly increase the risks of all-cause mortality, ATE, and non-fatal HF compared with the IVR treatment. The differences remained insignificant after adjusting for confounding factors, censored observations, and selection bias using multivariable time-to-event analyses with PS adjustment.

### Comparison with Other Studies

Our results were consistent with published RCTs, meta-analyses, and observational studies [16–18, 22–24, 27, 33, 38, 47–49], showing that patients treated with IVB had low rates of serious ocular and systemic adverse events. We found slightly higher rates of anti-VEGF-associated systemic events in the IVB group compared to the IVR group, but these differences were not statistically significant.

Compared to two RCTs conducted in nAMD patients in the US and the UK (CATT and IVAN) [22, 27], our study observed outcomes within a shorter period while participants overall were younger with lower rates of recent vascular events. We found similar proportions of patients experiencing SAEs including all-cause mortality and ATE in both IVB and IVR groups, and the frequencies were lower than in those head-to-head trials. The CATT study revealed a higher percentage of patients with at least one SSAE in the IVB group [39.9 vs 31.7%; adjusted risk ratio (RR) 1.30; 95% CI 1.07–1.57]. However, these events were not likely related to drug action on the VEGF pathway and the impact of different rates of TIA and MI history between the IVB and IVR groups at the baseline was not clearly explained [22]. A non-industry-funded RCT conducted by The Diabetic Retinopathy Clinical Research Network (DRCR.net) found similar risks of any Antiplatelet Trialists’ Collaboration Event (i.e. non-fatal MI, non-fatal stroke, or vascular death) after adjusting for age, sex, hemoglobin A1c level at the baseline, diabetes type and time since diagnosis at the baseline, insulin use, prior coronary artery disease (CAD), prior MI, prior stroke, prior TIA, prior HT, and smoking status among DME patients treated with aflibercept (5%), bevacizumab (8%), and ranibizumab (12%) [49].

Poku et al. [16] reviewed 22 RCTs and 67 observational studies with a minimum of 10 participants (sample sizes ranged from 11–27,962 IVB-treated patients) and reported low rates of SAEs but could not elucidate the relationship between IVB and those events. In comparison with studies included in this systematic review [16] and a safety review published in 2017 [33], our study found no excess ocular and systemic risks. Both reviews [16, 33] also highlighted the limitations of previous evidence. Randomized trials were underpowered to detect statistically significant differences in rare SAEs and excluded patients at high cardiovascular risk, causing limited generalizability. Most of the existing observational studies had low quality and limitations such as small sample sizes, ambiguous diagnostic criteria, and poor reporting of study outcomes. Our study was designed as a prospective multicenter cohort study and the sample size was larger than most of the previous observational studies. Most of these studies were descriptive studies with no comparator and had retrospective study designs. They also had small sample sizes and poor reporting on handling potential confounders except in a few of the large observational studies using healthcare databases in developed countries [33]. A large retrospective cohort study [47] using the Medicare database included 38,718 and 19,026 patients with nAMD in the IVB and IVR groups, respectively. This study found a significantly higher risk of stroke in the IVB group compared to the IVR group in the primary analysis. However, a higher SES might increase the probability of receiving IVR and is vulnerable to selection bias. After further adjustment for SES, there were no differences in the risks of mortality (aHR 1.10; 95% CI 0.85–1.41), MI (aHR 0.87; 95% CI 0.53–1.41), bleeding (aHR 1.01; 95% CI 0.80–1.28), and stroke (aHR 0.87; 95% CI 0.61–1.24) in the IVR group compared with the IVB group. A population-based nested case–control study [18] used linked data from Ontario’s healthcare databases (*n* = 91,378) and matched patients based on age, sex, history of outcomes, and DM. The results reaffirmed that there were no differences in risk of ischemic stroke (aHR 1.03; 95% CI 0.67–1.60), acute MI (aHR 1.23; 95% CI 0.85–1.77), VTE (aHR 0.92; 95% CI 0.51–1.69), and CHF (aHR 1.35; 95% CI 0.93–1.95) in the IVB group compared with the IVR group. Although these studies [18, 47] had large sample sizes and described their attempts to deal with potential confounding factors, they did not take the effect of repeated doses of drugs into account. Moreover, SAEs and death outside of hospitals were not captured using these databases and their results may not be generalized to the patient population not covered in the databases.

The multivariable parametric models in our study showed that the increase in the number of comorbid conditions was associated with increased risks of all-cause mortality, ATE, and non-fatal HF. This finding emphasizes the importance of taking these cardiovascular risk factors into account for high-risk patients.

We found that patients who received more than three injections of IVB or IVR had lower rates of all-cause mortality and ATE. These decreased risks disagreed with biological plausibility. If thromboembolic risk caused by systemic VEGF inhibition is associated with a dose-response relationship, the results in the present analysis do not support this assumption. This trend is similar to the pooled results from the CATT [26] and IVAN [27] studies. The group treated with less frequent injections either as-needed or via treatment discontinuation after three consecutive monthly injections had a significant 51% lower risk of death [pooled odds ratio (OR) 0.49; 95% CI 0.27–0.86] [27]. Etminan et al found no significant different risk of MI (adjusted RR 0.71; 95% CI 0.41–1.22) and stroke (adjusted RR 0.81; 95% CI 0.39–1.65) between nAMD patients who received a higher number of IVB injections and patients who received less than the median number of injections for each outcome [48].

In the case of ocular safety, the overall prevalence of endophthalmitis found in our study was 0.03% per injection (0.09% per person). This low rate was in line with findings of other studies conducted in the Thai setting [37, 39, 50], previous observational studies [16], and the CATT study [22]. We could not perform formal statistical tests to investigate the relationship between endophthalmitis and IVB preparation or other potential risk factors due to the limited number of events. In this study, there was one case that could be confirmed as postoperative endophthalmitis from cataract surgery. Thus, it was clear that this case was not related to either a drug-related or procedure-related complication. Moreover, we found that there was no occurrence of endophthalmitis in other patients from the same study site who received IVB from the same repackaged lot, the same dates of preparation and expiration after repackaging, and the same injection date and were injected by the same physicians at the same injection room as those endophthalmitis cases. Therefore, it might be assumed that the risk of endophthalmitis might be associated with patient-related factors rather than drug-related and procedure-related factors.

### Limitations of Study

First, this study was vulnerable to potential confounding and selection bias due to the nature of non-randomized studies. Statistical methods were conducted to account for this issue. Where the number of events was sufficient, multivariable analyses in conjunction with the PS method were performed to address any interference effects from well-established risk factors of cardiovascular disorders and imbalance of demographics and baseline characteristics. Second, our results represented only short-term safety profiles. We considered that 6 months of safety observation would be sufficient to capture the safety of IVB and IVR based on pharmacokinetic data [51] and time to develop SSAEs reported by previous studies [24, 52] together with opinion from retina specialists. Third, adverse events or other SAEs were beyond the scope of this study. Furthermore, we did not assess the safety of aflibercept, the VEGF trap, because it was introduced into Thailand approximately around the end of our data collection period. Lastly, the small number of participants in the IVR group and small number of events in this group led to imprecise estimates with wide 95% CIs, which did not allow us to conduct a comparative assessment for individual SSAEs of interest between IVB and IVR treatments. Moreover, none of the endophthalmitis cases was found in the IVR group. This study could not prove the absence of the potential difference in endophthalmitis rates between the IVB and IVR groups. This result should, therefore, be interpreted with caution.

### Conclusions and Policy Implications

This research provides safety evidence supporting the inclusion of off-label IVB into the Thai NLEM and the WHO EML. According to the nature of the observational study, our findings can be generalized to treatments for a wide variety of patient characteristics including patients at high risk in real clinical settings. Moreover, the results also demonstrated that both IVB and IVR have been used not only for nAMD and DME, but also for non-FDA approved indications in routine practice; however, IVR was likely to be prescribed according to FDA-approved indications. Since published RCTs were mainly conducted in patients with nAMD, DME, and RVO, the findings from this study may be used as supplementary evidence for the safety of IVB in other retinal conditions.

It also provides the information about endophthalmitis in the Thai context that differs from the clinical environment in developed countries. Moreover, the findings reveal the importance of proper management for patients at risk such as patient counseling or consideration of alternative treatments which do not increase the risk of such adverse events.

Apart from the safety issue, it could be clearly seen that the extent of off-label IVB usage was much higher than IVR (IVB 94% vs IVR 6%) and has been increasing since the NLEM policy was implemented. According to the NHSO’s report presented at The Prince Mahidol Award Conference 2016, the number of new IVB-treated patients with nAMD and DME in 2015 had increased by a factor of 1.6 compared to the number in 2013, and the cumulative number of these patients and total injections were 11,306 patients and 40,911 injections over 3 years after the policy launch.

As far as the NHI is concerned, CSMBS insurers received IVR treatment approximately 4.4 times more than IVB treatment. A possible reason might be related to the coverage of the pharmaceutical benefit packages as only the CSMBS provides full reimbursement for IVR therapy when the beneficiary has been diagnosed with FDA-licensed indications. In contrast, non-CSMBS patients must either pay out-of-pocket for this expensive drug or use alternative treatment options including IVB and non-pharmacological interventions. The number of IVR-treated patients in Thai public hospitals was small and may decrease over time because of its high cost, implementation of the NLEM policy, and use of aflibercept. Aflibercept is the newest FDA-approved anti-VEGF agent for treating nAMD, DME, and RVO and its cost in Thailand is as high as ranibizumab. Thus, IVB remains the most affordable option.

Based on the cumulative safety evidence and our findings, it may be concluded that the risks of IVB were low in terms of anti-VEGF-associated SAEs and endophthalmitis when properly compounded as a single dose for ocular use. The available evidence also supported the notion that IVB and IVR treatments had similar efficacy and safety profiles. It is likely that findings from further research with longer follow-up periods and larger sample sizes might not differ from currently available evidence.

## Electronic supplementary material

Below is the link to the electronic supplementary material.

Supplementary material 1 (PDF 59 kb)
