# Correction to: Real-World Safety of Intravitreal Bevacizumab and Ranibizumab Treatments for Retinal Diseases in Thailand: A Prospective Observational Study

**DOI:** 10.1007/s40261-019-00747-y

**Published:** 2019-01-19

**Authors:** Sermsiri Sangroongruangsri, Usa Chaikledkaew, Suthasinee Kumluang, Olivia Wu, Claudia Geue, Tanapat Ratanapakorn, Pattara Leelahavarong, Lily Ingsrisawang, Paisan Ruamviboonsuk, Wongsiri Taweebanjongsin, Janejit Choovuthayakorn, Apichart Singalavanija, Prut Hanutsaha, Kittisak Kulvichit, Thitiporn Ratanapojnard, Warapat Wongsawad, Yot Teerawattananon

**Affiliations:** 1 Social and Administrative Pharmacy Excellence Research (SAPER) Unit, Department of Pharmacy, Faculty of Pharmacy, Mahidol University, 447 Sri-Ayuthaya Road, Rajathevi, Bangkok, 10400 Thailand; 2 Health Intervention and Technology Assessment Program, Ministry of Public Health, Nonthaburi, Thailand; 3 Institute of Health and Wellbeing, University of Glasgow, Glasgow, UK; 4 Department of Ophthalmology, Khon Kaen University, Khon Kaen, Thailand; 5 Department of Statistics, Faculty of Science, Kasetsart University, Bangkok, Thailand; 6 Department of Ophthalmology, Faculty of Medicine, Rajavithi Hospital, Rangsit University, Bangkok, Thailand; 7 Mettapracharak Eye Institute, Mettapracharak (Wat Rai Khing) Hospital, Nakhon Pathom, Thailand; 8 Department of Ophthalmology, Faculty of Medicine, Chiang Mai University, Chiang Mai, Thailand; 9 Department of Ophthalmology, Faculty of Medicine Siriraj Hospital, Mahidol University, Bangkok, Thailand; 10 Department of Ophthalmology, Ramathibodi Hospital, Mahidol University, Bangkok, Thailand; 11 Department of Ophthalmology, Faculty of Medicine, Chulalongkorn University, Bangkok, Thailand; 12 Department of Ophthalmology, Phramongkutklao Hospital, Phramongkutklao College of Medicine, Bangkok, Thailand

## Correction to: Clinical Drug Investigation (2018) 38:853–865 10.1007/s40261-018-0678-5

The Open Access license, which previously read:

**Open Access**

This article is distributed under the terms of the Creative Commons Attribution-NonCommercial 4.0 International License (http://creativecommons.org/licenses/by-nc/4.0/), which permits any noncommercial use, distribution, and reproduction in any medium, provided you give appropriate credit to the original author(s) and the source, provide a link to the Creative Commons license, and indicate if changes were made.

Should read:

**Open Access**

This article is distributed under the terms of the Creative Commons Attribution 4.0 International License (http://creativecommons.org/licenses/by/4.0/), which permits unrestricted use, distribution, and reproduction in any medium, provided you give appropriate credit to the original author(s) and the source, provide a link to the Creative Commons license, and indicate if changes were made.

The original article has been corrected.

