# Supplementary material for: Real-World Safety of Intravitreal Bevacizumab and Ranibizumab Treatments for Retinal Diseases in Thailand: A Prospective Observational Study
Source: Clin Drug Investig. 2018 Aug 1;38(9):853–65. doi: 10.1007/s40261-018-0678-5 (PMC6153972; doi:10.1007/s40261-018-0678-5)
Supplement: Supplementary file 1 — Supplementary material 1 (PDF 59 kb) [file 40261_2018_678_MOESM1_ESM.pdf]

Electronic Supplementary Material (ESM)

Journal: Clinical Drug Investigation

Real world safety of intravitreal bevacizumab and ranibizumab treatments for retinal diseases in Thailand: a prospective observational study.

Sermsiri Sangroongruangsri[1], Usa Chaikledkaew[1*], Suthasinee Kumluang[2], Olivia Wu[3], Claudia Geue[3], Tanapat Ratanapakorn[4], Pattara Leelahavarong[2], Lily Ingsrisawang[5], Paisan Ruamviboonsuk[6], Wongsiri Taweebanjongsin[7], Janejit Choovuthayakorn[8], Apichart Singalavanija[9], Prut Hanutsaha[10], Kittisak Kulvichit[11], Thitiporn Ratanapojnard[12], Warapat Wongsawad[7], Yot Teerawattananon[2]

[1]Social and Administrative Pharmacy Excellence Research (SAPER) Unit, Department of Pharmacy, Faculty of Pharmacy, Mahidol University, Bangkok, Thailand.

[2]Health Intervention and Technology Assessment Program, Ministry of Public Health, Nonthaburi, Thailand.

[3]Institute of Health and Wellbeing, University of Glasgow, Glasgow, UK.

[4]Department of Ophthalmology, Khon Kaen University, Khon Kaen, Thailand.

[5]Department of Statistics, Faculty of Science, Kasetsart University, Bangkok, Thailand.

[6]Department of Ophthalmology, Faculty of Medicine, Rajavithi Hospital, Rangsit University, Bangkok, Thailand.

[7]Mettapracharak Eye Institute, Mettapracharak (Wat Rai Khing) Hospital, Nakhon Pathom, Thailand.

[8]Department of Ophthalmology, Faculty of Medicine, Chiang Mai University, Chiang Mai, Thailand.

[9]Department of Ophthalmology, Faculty of Medicine Siriraj Hospital, Mahidol University, Bangkok, Thailand.

[10]Department of Ophthalmology, Ramathibodi Hospital, Mahidol University, Bangkok, Thailand.

[11]Department of Ophthalmology, Faculty of Medicine, Chulalongkorn University, Bangkok, Thailand.

[12]Department of Ophthalmology, Phramongkutklao Hospital, Phramongkutklao College of Medicine, Bangkok, Thailand.

* Corresponding author: Usa Chaikledkaew

Email: usa.chi@mahidol.ac.th

Table 1 Comorbid conditions of patients in this cohort

Demographic variables	Ranibizumab (n= 379)	Bevacizumab (n= 5,975)
**Comorbidities**		
Diabetes		
no	231 (61.0%)	1,750 (29.3%)
yes	146 (38.5%)	4,212 (70.5%)
missing values	2 (0.5%)	13 (0.2%)
Hypertension		
no	123 (32.5%)	1,738 (29.1%)
yes	254 (67.0%)	4,223 (70.7%)
missing values	2 (0.5%)	14 (0.2%)
Dyslipidemia		
no	170 (44.9%)	2,451 (41.0%)
yes	207 (54.6%)	3,510 (58.7%)
missing values	2 (0.5%)	14 (0.2%)
Chronic kidney disease		
no	349 (92.1%)	5,132 (85.9%)
yes	29 (7.7%)	827 (13.8%)
missing values	1 (0.3%)	16 (0.3%)
Ischemic heart disease		
no	343 (90.5%)	5,576 (93.3%)
yes	34 (9.0%)	384 (6.4%)
missing values	2 (0.5%)	15 (0.3%)
Stroke		
no	353 (93.1%)	5,703 (95.5%)
yes	24 (6.3%)	257 (4.3%)
missing values	2 (0.5%)	15 (0.3%)

Data were presented as number (percentage).

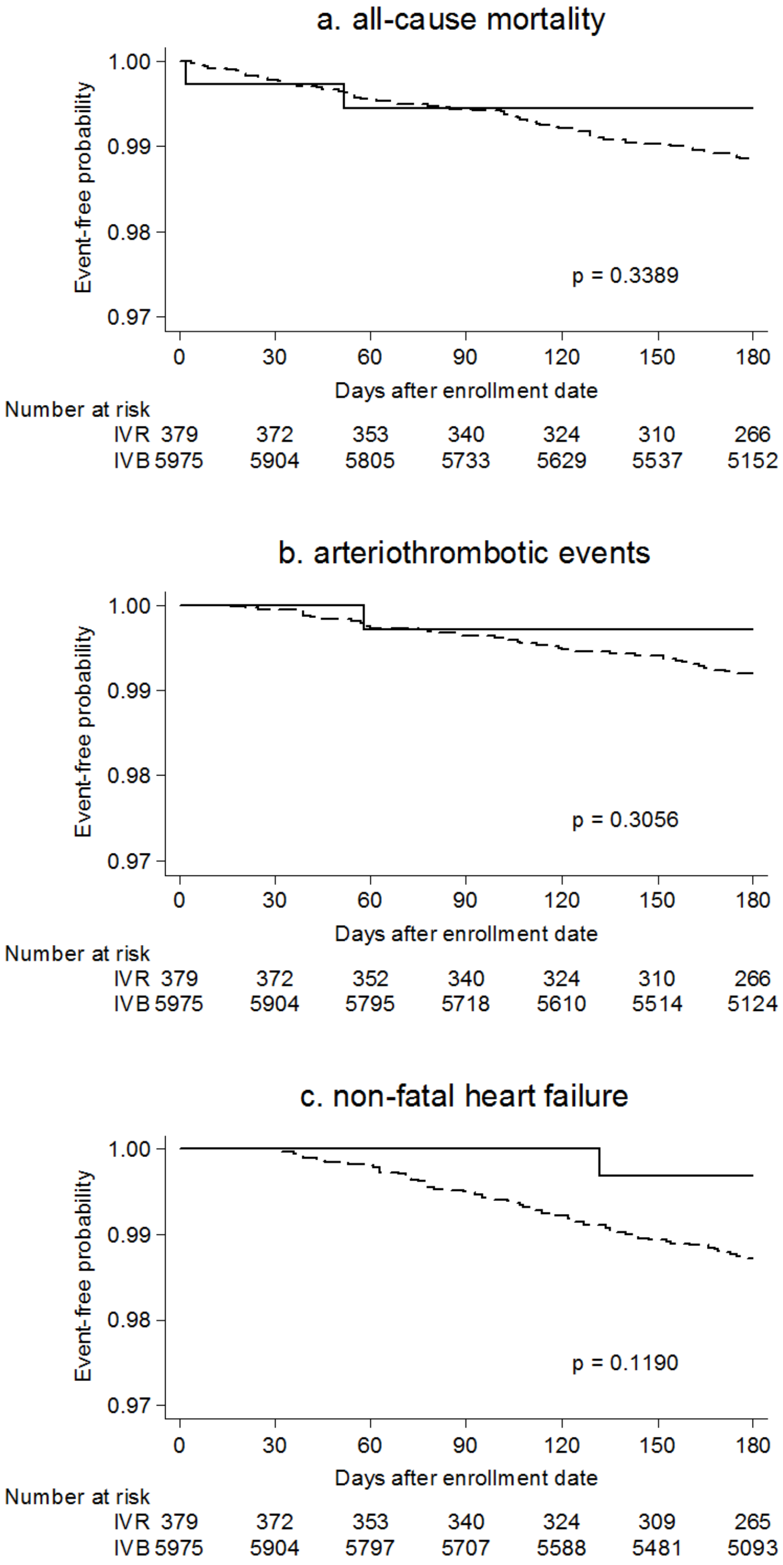

Fig. 1 Unadjusted Kaplan-Meier Estimates by treatment groups

(p-values for the log-rank test)
